# Omission of Axillary Surgery After Neoadjuvant Therapy in Her2-Positive Breast Cancer: Who Are the Candidates?

**DOI:** 10.3390/cancers17040562

**Published:** 2025-02-07

**Authors:** Omar Hamdy, Khalid Atallah, Alyaa R. Elsergany, Sara Atwa, Rana Abdo, Ali Zaher, Mostafa Abdelhakiem

**Affiliations:** 1 Surgical Oncology Department, Oncology Center, Mansoura University, Mansoura 35516, Egypt; khalid_atallah@mans.edu.eg; 2 Medical Oncology Unit, Oncology Center, Mansoura University, Mansoura 35516, Egypt; loloelsergany@mans.edu.eg (A.R.E.); saraatwa@mans.edu.eg (S.A.); mostafaabdelhakiem@mans.edu.eg (M.A.); 3 Mansoura University Hospitals, Mansoura 35516, Egypt; ranaelsayed35@std.mans.edu.eg; 4 Faculty of Medicine, Helwan University, Cairo 11813, Egypt; alizahir1998@gmail.com

**Keywords:** breast cancer, Her2, pathological complete response, sentinel lymph node biopsy, neoadjuvant therapy

## Abstract

**Background:** This study aimed to evaluate the lymph node (LN) response in Her2-positive breast cancer patients who received neoadjuvant therapy (NAT) and to discuss if axillary staging can be omitted in these patients. **Methods:** This is a retrospective cohort study including patients with Her2+ breast cancer at our center from March 2022 to September 2023. **Results:** The study included 139 patients in the final analysis; 69.1% of the patients had HR+/Her2+ disease while 30.9% had HR−/Her2+ disease. Most of the included patients (129 patients = 92.8%) had initially node-positive or suspicious disease. A total of 112 patients (80.6%) showed clinically negative axillary LNs (cN0) after NAT. All of them showed pathologically node-negative disease. When comparing the pathological node-positive vs. -negative groups, the following was found to be statistically significant: degree of Her2 positivity, number of anti-Her2 cycles, pre- and post-neoadjuvant LN status, and primary tumor radiological and pathological response. Also, all T1 tumors before NAT showed negative LNs after NAT, but it was statistically non-significant. **Conclusions:** Axillary staging may be omitted in Her2+ patients with cN0 after NAT, especially those with early tumors, strong Her2+ disease, cN0 before NAT, and those in whom the primary tumor achieved a complete clinical response.

## Simple Summary

The omission of axillary surgery is gaining popularity in selected groups of breast cancer patients. On the other hand, the exceptional nodal responses to neoadjuvant chemotherapy and dual Her2 blockage in Her2-positive breast cancer patients opens the way to the question of whether we can omit axillary surgery in such a group of patients or at least in a certain subgroup. This study aimed to evaluate the lymph node pathological status after neoadjuvant therapy in Her2+ breast cancer patients and to highlight the variables that can predict a pathological complete nodal response so that it can be expected without the need for surgical axillary staging

## 1. Introduction

The management of the axilla in breast cancer patients does not stop evolving. It started with performing axillary lymph node dissection (ALND) in all breast cancer patients, which was considered the standard of care in the staging and treatment. Then, sentinel lymph node biopsy (SLNB) replaced ALND as the standard of care in node-negative early breast cancer. However, the journey of evolution did not stop there [1,2,3,4,5]. More recently, SLNB has becoming increasingly common in the surgical management of breast cancer patients with locally advanced and even limited node-positive disease [1,2,6]. In addition, its role in breast cancer patients with node-positive disease that turned negative after neoadjuvant therapy also became a standard of care with or without targeted axillary dissection [4,7,8,9,10]. In the recent guidelines, neoadjuvant therapy became indicated in node-positive disease that is expected to convert to node-negative after NAT, so that the patients can undergo SLNB instead of ALND [11].

In addition, the omission of axillary staging has gained popularity in recent years in selected patient groups in whom either axillary staging is usually expected to be negative in almost all cases, or in whom the omission of axillary staging does not have an impact on the prognosis [12,13,14]. These patient groups include patients with DCIS who are prepared for breast-conserving surgery [5,11,15], elderly patients with luminal breast cancer [5,7,16], certain pathological types such as tubular carcinoma [17], and, most recently, patients with T1 tumors as reported by the SOUND trial [18].

On the other hand, neoadjuvant therapy has gained an expanding role in the management of breast cancer, not only in the locally advanced setting but also in patients with early breast cancer with pathological types that are expected to achieve a complete response, namely Human Epidermal growth factor receptor (Her)2-enriched and triple-negative subtypes [19]. The rates of tumor complete response are high in these types and the rates of nodal complete response are even higher [20]. The rate of axillary pCR in Her2-enriched tumors is reported to be up to 44.9% in those patients who received chemotherapy alone, 78.2% in those who received chemotherapy and single anti-Her2 blockage, and 80.2% in those who received chemotherapy and dual anti-Her2 blockage [21]. The best response is predicted when using chemotherapy + trastuzumab + pertuzumab [22]. This opens the question of whether we can omit axillary staging in such a category of patients.

In Egypt, after launching the national breast initiative [23], anti-Her2 drugs became available to Her2-positive breast cancer patients in the neoadjuvant setting. This opened the way to achieving much better responses in those patients at the tumor and lymph node levels.

The aim of this study was to evaluate the lymph node response in Her2-positive patients who received neoadjuvant chemotherapy and Her2 blockage in light of our financial and logistic capabilities, and to discuss if axillary staging can be omitted in these patients or at least regulated.

## 2. Materials and Methods

**Study design**: This was a retrospective cohort study

**Study population**: All patients who were diagnosed with Her2-positive breast cancer at Oncology Center Mansoura University during the period from March 2022 to September 2023 were included. Demographic, neoadjuvant, preoperative, operative, postoperative, pathologic, and oncologic follow-up data were retrieved from a prospectively maintained electronic database.

**Inclusion Criteria**: 1. Female patients with histological and radiological proof of non-metastatic breast cancer. 2. Age >18 years old. 3. Patients with invasive Her2 +ve breast cancer received neoadjuvant chemotherapy combined with single or dual Her2 blockage.

**Exclusion criteria**: 1. Patients with DCIS. 2. Patients with metastatic disease at the time of diagnosis. 3. Patients with locally advanced (T4b+) breast cancer. 4. Cases for whom anti-Her2 could not be delivered, either for cardiac disease or logistic issues.

**Study outcomes**: The aim of this study was to examine the axillary nodal response to neoadjuvant chemotherapy in combination with single or dual Her2-blockade (trastuzumab plus pertuzumab) and thus the possibility of omitting axillary surgery for those patients.

**Variables**:

The following variables were analyzed for each of the included patients:

- Patient characteristics: Age, BMI;
- Imaging: Imaging type, breast density, mass, site, size, focality, LN status, BIRADS, LN clipping;
- Pathological: pre-treatment LN biopsy, tumor type, hormonal receptor status, Ki67, Her score, luminal type, number of retrieved LNs after surgery, number of positive LNs;
- Neoadjuvant therapy: number of cycles, protocols, anti-Her2 type, number of cycles;
- Tumor response: RCB, Miller–Payne, tumor response, LN response;
- Surgery: tumor surgery type, LN surgery type;
- Adjuvant treatment: adjuvant radio, hormonal and target therapy.

**Treatment plan:**

1. **Chemotherapy protocols**: • Four cycles AC followed by four cycles of dose-dense paclitaxel with trastuzumab and pertuzumab. • Four cycles AC followed by weekly paclitaxel for 12 weeks with trastuzumab and pertuzumab. • Four cycles AC followed by four cycles of docetaxel with trastuzumab and pertuzumab • Docetaxel/carboplatin with trastuzumab and pertuzumab for 6 cycles. All the patients with residual disease after neoadjuvant therapy received TDM-1 (ado-trastuzumab).
2. **Breast and axillary examination and imaging after chemotherapy**: Breast ultrasound, mammogram, or MRI, and axillary ultrasound.
3. **Surgery** Breast-conserving surgery or mastectomy guided by the tumor response. Sentinel lymph node biopsy, targeted axillary dissection, or axillary lymph node dissection guided by the axillary response. Surgery was performed two to three weeks after finishing the course of chemotherapy.

**Statistical analysis**: Data were entered and analyzed using IBM-SPSS software (IBM Corp. Released 2020. IBM SPSS Statistics for Windows, Version 27.0. Armonk, NY, USA: IBM Corp.). Qualitative data are expressed as N and % and were compared by using chi-square, Fisher’s exact, or Fisher–Freeman–Halton exact tests, as appropriate. Quantitative data were initially tested for normality using Shapiro–Wilk’s test, with data being normally distributed if *p* > 0.050. The presence of significant outliers was tested by inspecting the boxplots. Quantitative data are expressed as median (Q1–Q3) and were compared by using the Mann–Whitney U-test. For any of the tests used, results were considered statistically significant if *p*-value ≤ 0.050. Also, an online calculator for diagnostic accuracy was used (https://onlineconfusionmatrix.com, accessed on 31 January 2025). Sensitivity = [true positive/(true positive + false negative)], specificity [true negative/(true negative + false positive)], positive predictive value = [true positive/(true positive + false positive)], negative predictive value = [true negative/(true negative + false negative)], accuracy = [(true positive + true negative)/all cases], and F1 score (a better measure than accuracy) = [(2 × true positive)/(2 × true positive + false positive + false negative).

The manuscript was prepared following the STROBE guidelines for reporting observational studies.

## 3. Results

This study involved 159 patients with Her2-positive breast cancer who received neoadjuvant chemotherapy and anti-Her2 therapy from March 2022 until September 2023. Out of these 159 cases, 20 patients were excluded because of missed data, not receiving anti-Her2, lost follow-up, escaping surgical intervention, or developing metastasis, so 139 patients were included in the final analysis. The basic clinical and demographic data of the included patients are summarized in Table 1. The median age of the included patients was 50 years, ranging from 24 to 73 years, while the median BMI was 32.5 kg/m^2^, ranging from 17.2 to 54.6 kg/m^2^.

Most of the included patients (97.8%) were evaluated initially by mammography, while MRI was performed for 31.7% of the patients. Breast imaging revealed that most of the included patients had ACR-dense B and C breasts (56.8% and 25.9%, respectively). Most tumors were unifocal (60.4%) and in the upper outer quadrant (55.4%). The median tumor diameter was 3.1 cm. Most of the patients had invasive ductal carcinoma (97.2%). Nearly two-thirds (69.1%) had hormonal receptor (HR)+/Her2+ disease, while about one-third (30.9%) had HR−/Her2+ disease.

Most of the included patients (129 patients = 92.8%) had node-positive or suspicious disease at the time of diagnosis, but only 26 patients (18.7%) were confirmed by US-guided biopsy. All the patients received neoadjuvant chemotherapy + anti-Her2 therapy (dual anti-Her2 in 135 patients) followed by surgery. The number of anti-Her2 cycles varied according to the planned protocol as well as administrative paperwork issues, but most of the patients (82.8%) received 2–4 cycles.

Following neoadjuvant therapy, all the patients underwent breast and axillary surgery. Preoperatively, the patients were reevaluated by breast and axillary imaging. A total of 112 patients (80.6%) showed clinically and radiologically negative axillary lymph nodes after NAT. After surgical axillary staging (ALND performed for 100 patients or SLNB performed for 39 patients following the surgeon’s decision), these findings were concordant with the pathological evaluation of lymph nodes, as 112 patients showed node-negative disease and 27 patients showed node-positive disease. All the patients treated by SLNB only had node-negative disease, whereas regarding the patients who underwent ALND, 73 showed node-negative disease while 27 showed node-positive disease.

Regarding the final pathological LN status, when comparing the two groups (pathological node-positive vs. -negative), the following factors were found to be statistically significant (Table 2):

- Degree of Her2 positivity (Her2+ 3 tumors were more likely to develop nodal pCR);
- Type of anti-Her2 therapy (dual blockage more likely to develop nodal pCR);
- Pre-neoadjuvant LN status by imaging (all the radiologically negative LNs turned out to be pathologically negative);
- Post neoadjuvant LN status by imaging (all the radiologically negative LNs turned out to be pathologically negative);
- Primary tumor radiological response (all the patients who had cCR also had nodal pCR);
- The degree of pathological tumor response (all the patients with RCB0 or Grade 5 Miller–Payne had nodal pCR);
- All T1 primary tumors before NAT showed negative LNs after NAT, but it was statistically non-significant.

The diagnostic accuracy of the significant variables in predicting LN positivity is summarized in Table 3.

Binary logistic regression was run to ascertain the effects of Her2 score (3 vs. 2), target therapy type (trastuzumab + pertuzumab vs. trastuzumab alone), chemotherapy (AC plus Taxan vs. Taxan alone), and anti-Her2 cycles (4–5 vs. 1–3) on the likelihood of occurrence of pathologically negative axillary LNs (Table 4).

In univariable analysis, both Her2 score and target therapy type were significant predictors of pathologically negative axillary LNs.

In multivariable analysis, both Her2 score and target therapy were significant independent predictors of pathologically negative axillary LNs. The model was statistically significant (χ2 [4] = 13.916, *p* = 0.008). The model correctly classified 82.7% of cases with 29.6% sensitivity, 95.5% specificity, 61.5% PPV, and 84.9% NPV (TP, TN, FP, and FN cases were 8, 107, 19, and 5, respectively). Participants with scores 3 vs. 2 and participants who received trastuzumab plus pertuzumab vs. trastuzumab alone had 5.1 and 14.4 times higher odds of exhibiting pathologically negative axillary LNs.

## 4. Discussion

“The best doctor gives the least medicines”. This well-known role can be applied in all fields of medicine as long as efficacy and safety are guaranteed. In the oncology field, shifting from a more aggressive form of treatment to a less aggressive one needs solid confirmation that this shift does not compromise the oncological outcomes or affect disease-free survival and overall survival [24,25,26,27,28]. In the field of axillary surgery in breast cancer patients, the continuous de-escalation and minimizing the value of aggressive axillary surgeries have been supported by many studies that provided enough evidence for the currently applied practice [5,10,29,30,31,32]. The findings of our research provide a step forward toward the omission of axillary surgery for breast cancer patients with exceptional responses after neoadjuvant therapy. In addition, this will not lead to overtreatment of the patients, as the decision whether to receive adjuvant radiotherapy or not is based on the type of surgery and the initial tumor volume and nodal status [11].

The addition of anti-Her2 target therapy to neoadjuvant chemotherapy protocols led to a marked increase in the rates of pathological complete response in Her2+ breast cancer. It opened the way even to ask if breast surgery itself can be declined [19]. Furthermore, previous studies confirmed that the rates of axillary pCR are higher than those of the primary tumors, especially when dual anti-Her2 treatment is used. Our results confirmed the same findings. While most of the included patients (129 patients = 92.8%) had node-positive or suspicious disease at the time of diagnosis, only 27 patients (19.4%) showed pathological lymph node infiltration after neoadjuvant therapy. This means that our cohort—even with the small number of cycles received by some patients—showed a complete pathological axillary response in 79.1% of patients, in line with previously published studies [33].

These results open the way to question if we can select, at least, a subgroup of patients in whom axillary staging can be omitted. The selection should be based on clear criteria that can predict the status of axillary lymph nodes without the need for surgical lymph node excision. Determining how to select those patients has been the core of many recent and ongoing trials, such as the EUREAST-01 trial [34], ASICS trial [35], OPTIMIST trial, [36] and ASLAN trial [37]. The results of our study showed that the following factors could independently predict complete nodal response: the pre-neoadjuvant LN status by imaging, the primary tumor complete clinical response, and the axillary LN complete clinical response. Also, all the patients who had T1 tumors before NAT showed a complete axillary pathological response. In addition, the Her2 expression status and the type of anti-Her2 blockage showed a significant correlation with achieving axillary pCR (*p*-value = 0.024 and 0.023, respectively).

The first finding in our study was that all the patients with cN0 before neoadjuvant therapy had a pathologically negative axilla after neoadjuvant therapy. Although the number of patients in this category was small (10 patients only), these findings show similar outcomes to the study by Samiei et al. [38], who reported a 100% correlation in the ER-Her2+ subtype and 98.4% correlation in the ER + Her2+ subtype provided that breast pCR was achieved.

Another significant finding is that all the patients with T1 disease had a pN0 disease after neoadjuvant therapy. This is relatively similar to the findings reported by Tadros et al. [29], who reported a pN0 for 100% of the patients with T1N0 HER+ tumors and pN0 of 76.9% and 71.4% of the patients with T1N1 HER+ tumors who achieved and did not achieve breast pCR, respectively.

In our study, an absolute correlation was found between the tumor cCR and the axillary pCR. This compares with previously published studies [29,39], which reported that the rate of axillary nodal positivity with tumor complete response is exceedingly rare. This opens the way towards omitting SLNB in patients who achieve primary tumor complete response. This absolute correlation was not reported in other studies, such as the study by Guo et al. [40], who reported a total axillary pCR of about 80% of those who achieved tumor pCR. This may be explained by the fact that most of the patients in this study received single anti-Her2 treatment, contrary to our cohort in which most patients received dual anti-Her2 treatment. Other studies confirmed this absolute correlation only in initially cN0 disease [29,39]

Receiving dual anti-Her2 treatment is reported in our study to be significantly correlated with axillary pCR. These results have been confirmed by the previously published literature, including a recent network meta-analysis by Luo et al. [22], who reported that dual Her2 blockage is significantly better than single blockage in achieving pCR.

Another significant finding in our study is that all the patients who were reported clinically and radiologically to have negative lymph nodes were also pathologically proven to have an axillary complete pathological response. To our knowledge, this may be the first study to report such a result. Previous studies, like that reported by Qiu et al., reported the negative predictive value of axillary ultrasound in cT ≤ 3 cm, cN0, and Her2-positive tumors to be 78.5% and 89.6% if one lymph node metastasis was considered negative, and 93.3% if one lymph node metastasis was considered negative.

Regarding the Her2 expression status, our results showed that Her2+ 3 tumors are more likely to achieve axillary pCR than Her2+ 2 tumors (*p*-value = 0.024). This compares with the findings of Mou et al. [33], who reported the same findings with a *p*-value of 0.049 in their study, which included 215 patients with Her2-positive disease, and with the results of Chen et al. [41], who reported the same findings with a *p*-value of 0.015 in their study, which included 224 patients with Her2-positive disease.

Our study has limitations. As a retrospective study, it is liable to selection bias. Other limitations include the heterogeneity in the chemotherapy protocols, the non-equality of the numbers of anti-Her2 therapy cycles received by the patients, and the heterogeneity in the axillary staging procedures and surgeons. On the other hand, the strengths of our study include that the data are prospectively maintained in our center’s electronic database, the number of included patients was not small, and the axillary staging procedures were decided by the surgeons’ own decisions, making it an experience similar to everyday practice.

## 5. Conclusions

Axillary staging may be omitted in Her2+ breast cancer patients who show clinically node-negative disease after neoadjuvant chemotherapy combined with dual anti-Her2 therapy, especially for those with early tumors, strong Her2+ disease, clinically negative lymph nodes before neoadjuvant therapy, and those in whom the primary tumor achieved a complete clinical response. We can suggest that, at least, the patients with cT1-2N0 tumors and breast CR are candidates for the omission of axillary staging. Further multicenter trials are needed to confirm the findings of this study. In addition, prospective randomized controlled multicenter trials are needed to evaluate the nodal recurrence rates in such a category of patients when axillary staging is declined.

## Figures and Tables

**Table 1:** Clinical and demographic parameters of the studied cases.

Characteristic	N/Median	%/Q1–Q3
Age	50	24–73
BMI	32.5	17.2–54.6
Imaging		
Mammogram	136	97.8%
MRI	44	31.7%
Breast density (ACR)		
A	15	10.8%
B	79	56.8%
C	36	25.9%
D	9	6.5%
Focality		
Unifocal	84	60.4%
Multifocal	33	23.7%
Multicentric	22	15.8%
Tumor site		
Upper outer quadrant	77	55.4%
Lower outer quadrant	15	10.8%
Upper inner quadrant	18	12.9%
Lower inner quadrant	15	10.8%
Central	14	10.1%
BIRADS		
3	2	1.4%
4	46	33.1%
5	91	65.5%
Largest diameter by imaging (mm)	31	8–100
Tumor size (T stage)		
T1	8	5.8%
T2	83	59.7%
T3	21	15.1%
T4	27	19.4%
Clinical/Radiological LN status (before NAT)		
Positive	129	92.8%
N3	10	7.2%
N2	16	74.1%
N1	103	11.5%
Negative	10	7.2%
Tumor biopsy type		
Core needle biopsy	138	99.3%
Excisional	1	0.7%
Pathological tumor type		
Invasive ductal carcinoma	136	97.8%
Invasive lobular carcinoma	2	1.4%
Invasive micro-papillary carcinoma	1	0.7%
Molecular profile		
Estrogen receptor:		
Positive	84	60.4%
Negative	55	39.6%
Progesterone receptor		
Positive	82	59%
Negative	57	41%
Degree of Her2 positivity		
Positive +2	10	7.2%
Positive +3	129	29.8%
Ki67	40	5–95
Luminal type		
Her2-enriched	43	30.9%
Luminal B	96	69.1%
LN biopsy before NAT		
Yes	26	18.7%
No	113	81.3%
LN clipping before NAT		
Yes	7	5%
No	132	95%
Type of chemotherapy		
AC + Taxane	132	95%
Taxane	7	5%
Type of target therapy		
Trastuzumab + Pertuzumab	135	97.1%
Trastuzumab	4	2.9%
Number of neoadjuvant anti-Her2 cycles		
1	17	12.2%
2	50	36%
3	29	20.9%
4	36	25.9%
5	7	5%
Post-NAT LN status (Radiological)		
Positive	27	19.4%
Negative	112	80.6%
Surgery type		
Breast-conserving surgery	59	42.4%
Mastectomy	80	57.6%
Axillary staging type		
Axillary LN dissection	100	71.9%
Sentinel lymph node biopsy	39	28.1%
Response		
Complete	87	62.6%
Partial	51	36.7%
No response	1	0.7%
Residual cancer burden (RCB) (Pathological)		
0	87	62.6%
1	14	10.1%
2	26	18.7%
3	11	7.9%
4	1	0.7%
The Miller–Payne grading system (Pathological)		
Grade 1	4	2.9%
Grade 2	8	5.8%
Grade 3	24	17.3%
Grade 4	16	11.5%
Grade 5	87	62.6%
Number of retrieved nodes	10	1–31
Number of positive nodes	0	0–9
Axillary lymph node pathology		
Positive	27	19.4%
Negative	112	80.6%
Adjuvant radiotherapy		
Yes	121	87.1%
No	18	12.9%

Notes: Categorical data are presented as N and %, while numerical data are presented as median (Q1–Q3).

**Table 2:** Comparison between pathological node-positive and -negative disease after NAT.

Characteristic	Node-Negative	Node-Positive	*p* Value
Age	50 (24–73)	50 (30–72)	0.873
BMI	32.8 (17.2–54.6)	30.5 (22.2–53.5)	0.816
Breast density (ACR)			0.936 ^FFH^
A	12 (8.6%)	3 (2.2%)	
B	64 (46%)	15 (10.8%)	
C	28 (20.1%)	8 (5.8%)	
D	8 (5.8%)	1 (0.7%)	
BIRADS			**0.037 ^FFH^**
3	1 (0.7%)	1 (0.7%)	
4	42 (30.2%)	4 (2.9%)	
5	69 (49.6%)	22 (15.8%)	
Largest diameter by imaging (mm)	32 (8–100)	31 (19–80)	0.729
Tumor size (T stage)			0.133 ^FFH^
T1	8 (5.8%)	0 (0%)	
T2	70 (50.4%)	13 (90.4%)	
T3	15 (10.8%)	6 (4.3%)	
T4	19 (13.7%)	8 (5.8%)	
Radiological LN status (before NAT)			0.223 ^FFT^
N3	9 (6.5%)	1 (0.7%)	
N2	11 (7.9%)	5 (3.6%)	
N1	82 (59%)	21 (15.1%)	
N0	10 (7.2%)	0 (0%)	
Molecular profile			
Estrogen receptor:			0.890 ^X^
Positive	68 (48.9%)	16 (11.5%)	
Negative	44 (31.7%)	11 (7.9%)	
Progesterone receptor			0.975 ^X^
Positive	66 (47.5%)	16 (11.5%)	
Negative	46 (33.1%)	11 (7.9%)	
Degree of Her2 positivity			**0.024 ^FET^**
Positive +2	5 (3.6%)	5 (3.6%)	
Positive +3	107 (77%)	22 (15.8%)	
Ki67	40 (10–90)	45 (5–95)	0.447
Luminal type			0.870 ^X^
Her2-enriched	35 (25.2%)	8 (5.8%)	
Luminal B	77 (55.4%)	19 (13.7%)	
Type of chemotherapy			0.621 ^FET^
AC + Taxan	107 (77%)	25 (18%)	
Taxan	5 (3.6%)	2 (1.4%)	
Type of target therapy			**0.023 ^FET^**
Trastuzumab + Pertuzumab	111 (79.9%)	24 (17.3%)	
Trastuzumab	1 (0.7%)	3 (2.2%)	
Number of neoadjuvant anti-Her2 cycles			0.515 ^FFH^
1	13 (9.4%)	4 (2.9%)	
2	40 (28.8%)	10 (7.2%)	
3	21 (15.1%)	8 (5.8%)	
4	32 (23%)	4 (2.9%)	
5	6 (4.3%)	1 (0.7%)	
Number of neoadjuvant anti-Her2 cycles			0.120 ^X^
(Grouped)			
1–3	74 (53.2%)	22 (15.8%)	
4–5	38 (27.3%)	5 (3.6%)	
Post-NAT LN status (Radiological)			**<0.001 ^X^**
Positive	0 (0%)	27 (19.4%)	
Negative	112 (80.6%)	0 (0%)	
Primary tumor response			**<0.001 ^FFH^**
Complete	87 (62.6%)	0 (0%)	
Partial	25 (18%)	26 (18.7%)	
No response	0 (0%)	1 (0.7%)	

Notes: Categorical data are N (%) and numerical data are median (Q1–Q3). Sig. = statistical significance (*p*-value). The tests of significance are the Mann–Whitney U-test for numerical data, and the chi-square test (^x^), Fisher’s exact test (^FET^), and Fisher–Freeman–Halton exact test (^FFH^) for categorical data. The bold is of statistical significance.

**Table 3:** Diagnostic accuracy of significant characteristics in predicting positive vs. negative LN.

Characteristic	Sensitivity	Specificity	PPV	NPV	Accuracy	F1 Score
Her2 score (3 vs. 2)						
Overall	18.5%	95.5%	50%	83%	80.6%	27%
Chemotherapy protocol						
AC + Taxan	20%	96.3%	55.56%	96.2%	81.82%	29.4%
Taxan	0%	80%	0%	66.7%	57.14%	0%
Anti-Her2 cycles						
1–3	22.7%	96%	62.5%	80.7%	79.2%	33.3%
4–5	0%	94.7%	0%	87.7%	83.7%	0%
Type of target therapy (T vs. T/P)						
Overall	11.1%	99.1%	75%	82.2%	82%	19.4%
Chemotherapy protocol						
AC + Taxan	12%	99.1%	75%	82.8%	82.6%	20.7%
Taxan	0%	100%		71.4%	71.4%	0%
Anti-Her2 cycles						
1–3	13.6%	98.7%	75%	79.4%	97.2%	23.1%
4–5	0%	100%		88.4%	88.4%	0%
Rad. Pre-NAT-LN status (+ve vs. −ve)						
Overall	100%	9%	20.9%	100%	26.6%	34.6%
Chemotherapy protocol						
AC + Taxan	100%	9%	20.5%	100%	26.5%	34%
Taxan	100%	0%	28.6%		28.6%	44.4%
Anti-Her2 cycles						
1–3	100%	10.8%	25%	100%	31.4%	40%
4–5	100%	5.3%	12.2%	100%	16.3%	21.7%
Rad. Post-NAT LN status Positive vs. negative	100%	100%	100%	100%	100%	100%
T stage (T2–4 vs. 1)						
Overall	100%	7.1%	20.6%	100%	25.2%	34.2%
Chemotherapy protocol						
AC + Taxan	100%	7.5%	20.6%	100%	25%	33.5%
Taxan	100%	0%	28.6%		28.6%	44.4%
Anti-Her2 cycles						
1–3	100%	1%	24.4%	100%	29.2%	39.3%
4–5	100%	5.2%	12.2%	100%	16.3%	21.7%

Notes: T/P = trastuzumab + pertuzumab. T = trastuzumab. Rad = radiological. PPV = positive predictive value. NPV = negative predictive value.

**Table 4:** Predictors of pathologically negative axillary LN.

Predictor Variable	Univariable		Multivariable	
	COR (95% CI)	Sig.	AOR (95% CI)	Sig.
Her2 score		0.019		0.017
Score 2	r(1)		r(1)	
Score 3	4.9 (1.3–18.2)		5.1 (1.3–19.5)	
Target therapy		0.025		0.025
Trastuzumab	r(1)		r(1)	
Trastuzumab + Pertuzumab	13.9 (1.4–139.2)		14.4 (1.4–148.3)	
Chemotherapy		0.535		0.408
Taxan	r(1)		r(1)	
AC + Taxan	1.7 (0.31–9.3)		2.1 (0.35–12.9)	
Anti-Her2 cycles		0.127		0.222
1–3	r(1)		r(1)	
4–5	2.3 (0.79–6.4)		2 (0.66–6.1)	

Notes: COR = crude odds ratio. AOR = adjusted odds ratio. Sig. = statistical significance (*p*-value). 95% CI = 95% confidence interval. r(1) = reference category.

## Data Availability

All the clinical, radiological, and pathological data used in this manuscript are available on the Mansoura University medical system (Ibn Sina Hospital Management System). http://srv137.mans.edu.eg/mus/newSystem/, accessed on 31 January 2025.
